# Practical approaches of PULSE Racing in training their athlete for the Cybathlon Global Edition Functional Electrical Stimulation bike race: a case report

**DOI:** 10.1186/s12984-023-01143-6

**Published:** 2023-03-03

**Authors:** Heleen Docter, Katja Podvinšek, Sander Koomen, PULSE Racing III, Birgit E Kaman, Ilona Visser, Niek Klunder, Anneloes van den Berg, Laurien Bellens, Chrétine Wijnbelt, Sterre Groot

**Affiliations:** 1 Department of Human Movement Sciences, Faculty of Behavioural and Movement Sciences, Vrije Universiteit Amsterdam, Van Der Boechorststraat 9, 1081 BT Amsterdam, The Netherlands; 2 Amsterdam Movement Sciences, Amsterdam, The Netherlands

**Keywords:** FES cycling, Spinal cord injury, Race preparation, Cybathlon, Case report

## Abstract

During the Cybathlon Global Edition 2020, athletes compete in a Functional Electrical Stimulation (FES) bike race. In this event, athletes with a spinal cord injury cover a distance of 1200 m on an adapted bike by using electrostimulation to activate their leg muscles in order to evoke a pedalling movement. This report reviews the training regimen, as designed by the PULSE Racing team, and the experience of one athlete in preparation for the Cybathlon Global Edition 2020. The training plan was designed to vary exercise modes in order to optimize physiological adaptations and minimize monotony for the athlete. Additional constraints due to coronavirus pandemic, e.g., postponement of the Cybathon Global Edition and modification from a live cycling track to a virtual stationary race, along with the health concerns of the athlete, e.g. unwanted effects from the FES and bladder infection, required creativity to ensure an effective and safe training protocol. The individual needs of the athlete and task requirements for the FES bike race made the design of a suitable training programme challenging, emphasizing the importance of monitoring. Several objective and subjective measures to assess the athlete’s health and progress are presented, all with their own advantages and disadvantages. Despite these limitations, the athlete achieved a gold medal in the FES bike race Cybathlon Global Edition 2020 through discipline, team collaboration and the athlete’s own motivation.

## Background

The coronavirus disease 2019 (COVID-19) pandemic affected the lives of people worldwide, among which those with a pre-existing disability. Physical activity of people with disabilities, among which people with a spinal cord injury (SCI), significantly decreased in a very short time [1]. However, the PULSE Racing team and their athlete, Sander Koomen, proved their flexibility and capacity to adjust to the new COVID-19 situation, by developing and adhering to a successful training regimen to win gold in the Cybathlon Global Edition 2020 functional electrical stimulation (FES) bike race. The Cybathlon Global Edition 2020 is an international competition hosted by the Cybathlon, a non-profit project of the Swiss Federal Institute of Technology Zurich. The competition is organized to stimulate the development of technologies which assist the everyday life of people with disabilities. The FES bike race is one of the six disciplines of the Cybathlon Global Edition 2020. In this event, a recumbent tricycle is equipped with ankle orthoses to support the ankle and lower leg of the athlete. The use of FES enables paralyzed cyclists to perform leg cycling on an adapted bike by activating leg muscles in a specific pattern to elicit a pedalling movement. In the original format, athletes cover 1200 m on an oval indoor racetrack while being propelled by their externally stimulated leg muscles. Due to the gathering restrictions during COVID-19 pandemic, Cybathlon Organising Committee was forced to postpone the race and develop a safer format for competition. Technology provided new opportunities and an online racing platform was developed. During the Cybathlon Global Edition 2020, the recumbent bikes of the FES bike race were placed on stationary, direct drive, smart bike trainers to cover the 1200 m race online [2]. Besides the need for technological adaptations, the athlete required flexibility to adjust to the new racing format. The PULSE Racing team and their athlete modified training and optimized the bike set-up to excel in this redesigned FES cycling configuration.

PULSE Racing was the winning team in the FES bike race Cybathlon Global Edition 2020. As the mission of PULSE Racing is to make FES more accessible in the Netherlands, participating in the Cybathlon Global Edition 2020 generates a new platform of competitive cycling for people with SCI and maximize performance of the users. Thus, the purpose of this brief report is to give an overview of the preparations of PULSE Racing team towards the Cybathlon Global Edition 2020 and insight into solving challenges encountered during the preparation process. In line with our objectives for the paper, our athlete, Sander Koomen, will provide a perspective of the athlete experience during the training programme.

## Methods

Improving and optimizing the training strategy received much attention in PULSE Racing’s preparation towards the Cybathlon. The training intervention plan required information about the task and the physical capabilities of the athlete. This information was linked to physiological performance determinants, current strengths and points for improvement to define training objectives. Several long and short term goals were formulated according to the Specific, Measurable, Achievable, Relevant and Time-bound (SMART) principle to keep the athlete and team motivated [5]. The goals were adjusted over time, depending on the progress of the athlete.

### Characteristics of the participant

The characteristics of the athlete, i.e., the level and duration of SCI, are important to consider for elaboration of the training objectives. Muscle disuse (> 2 years) in the long term leads to atrophy of all fibre types and a loss of oxidative capacity [3]. As a result of these changes, the people with a SCI have a 70–80% lower muscle force, increased muscle fatiguability and increased contractile speed of muscle fibres [3, 4]. The degree to which muscles are affected after SCI is dependent on many factors such as the level of SCI, duration of SCI, age of the individual and general fitness of the individual. Variation of these components makes it important that training be specifically adapted to SCI athlete. Our athlete’s characteristics are summarized in Table 1. Diagnostic assessment (ASIA impairment score (AIS) and functioning) was performed by a qualified medical assessor and in agreement with the organisation of the Cybathlon Global Edition 2020. The first application of FES on the leg muscles happened in 2010. FES was used systematically from 2017.

**Table 1:** Athlete characteristics

Lesion hight	Lesion duration (y)	VO_2 max_ (mL/kg/min)	AIS score	Sensory function	Motor function
T5	27	33	A	T6 (R) T8 (L)	T5 (R) T5 (R)

*VO*_*2 max*_ maximal oxygen consumption (determined during hand biking 22-01-2019), *AIS* ASIA impairment scale, *R* right, *L* left

### Task requirements

The main goal was to cycle as fast as possible during the Cybathlon Global Edition 2020. The athletes have three trials to complete the 1200 m online race as fast as possible. The team with the fastest time over the three trials wins the race. From a physiological perspective, task requirements of the FES bike race resemble the physiological needs in track cycling, as the duration of the race is similar to the individual pursuit of able-bodied track cyclists. The current world record for the men’s individual pursuit is 4:01.934 min over a distance of 4000 m [6]. As for the individual pursuit in track cycling, fulfilment of the FES bike race requires a combination of sprint and endurance capacity. The physiological determinants for endurance and sprint performance (relative to body weight) are dissimilar [7] and training adaptations to strength and endurance training are contradictory [8]. Therefore, our training objectives focused on improving muscle fascicle length and enhancing capillarisation to address both sprint and endurance performance [7].

### Training programme

Evoking favourable physiological adaptations, i.e., enhanced capillarisation and increased muscle fascicle length, requires an extensive plan. Macro-, meso- and micro-plan were used to include all aspects in the training of the athlete. One year before the proposed date, the training load was planned at the macro level. The first phase (base training) occupied approximately eight months. The main focus of the training sessions was to improve the endurance capacity of the athlete at whole body level and muscle level. Although we were not directly measuring the fibre type composition, we attempted to induce a shift towards more type I fibres and increase capillarisation to improve endurance performance. Animal studies have shown that a period of intensive electrical stimulation could induce a switch to a slower muscle fibre phenotype [9]. The first months of the base training phase allowed development of personalized stimulation characteristics and get used to these adjustments. When the Cybathlon approached, the regiment shifted to specific training sessions that were more explosive. The aim of the specific training phase was to improve the power output of the athlete and thereby increase velocity. The last weeks before the Cybathlon Global Edition 2020, the training load was reduced to allow recovery. We aimed for a taper period of 14 days in which the training volume was reduced to 40–60% [10]. The remaining training sessions were designed to be intensive to provide a strong stimulus for maintenance of the fitness, while allowing the body plenty of time to recover [10].

After establishing the macro-plan, each phase was split into groups of 3–4 consecutive weeks. The plan at the meso level allows for application of the principle of supercompensation. Intermediate, high intensity and recovery weeks were included in periods of three to four weeks. High intensity weeks were, for example, intense strength blocks with two to three training sessions a day to disturb homeostasis of several processes in the muscular and cardiorespiratory system (i.e., lactate shuttle system, glycogen depletion). Each period, the load increased to induce progressive overload. One should note that intense weeks can be quite time consuming and fatiguing (i.e., 12 h of training, high stimulation frequency, adding weights). Intense weeks are followed by recovery weeks in which the athlete was given the opportunity to recover both mentally and physically from the training sessions (i.e., 6 h of training, low stimulation frequency, no additional weights). The goal of the reduction in training volume and intensity was to evoke supercompensation. The final 18 weeks before the Cybathlon Global Edition 2020 can be found in Table 2.

**Table 2:** Example of training programme at macro and meso level

Weekly countdown	Phase (macro level)	Average weekly training load (meso level)
18	Phase 1 base training	Recovery
17		Intermediate
16		High intensity
15		Recovery
14		Intermediate
13		High intensity
12		Recovery
11		Intermediate
10		High intensity
9	Phase 2 specific training	Recovery
8		Intermediate
7		High intensity
6		Recovery
5		High intensity
4		Intermediate
3		High intensity
2	Tapering	Tapering
1		Tapering

Micro-plan includes a plan of the training session within each week, based on the Frequency, Intensity, Time and Type (FITT) and polarized training principles. The FITT principle states that the frequency, intensity, time and type of training should favour the desired physiological adaptation [11]. The total training stimulus (frequency, intensity and time) should be strong enough to disrupt the homeostasis. For each parameter, the optimal total training stimulus requires a slightly different combination to evoke favourable adaptation. The general recommendations for training the cardiovascular system, muscle strength and muscle endurance in clinical practice (Table 3) [12] can be used as guidance, but one should be aware that paralyzed muscles have minimal activation levels throughout the day. Some translational signalling molecules return to baseline levels one to two hours after muscle activation [13], which might indicate that frequent muscle activation could be favourable to encourage an anabolic environment in paralyzed muscles. Therefore, some days included more than one training session and rest days included a short period of stimulation at a low frequency and amplitude.

**Table 3:** Guidelines for training the cardiovascular system, muscle strength and muscle endurance

	Cardiovascular system	Muscle strength	Muscle endurance
Frequency	3–5 sessions	2–3 sessions	2–3 sessions
Intensity	40–85% of VO2max Between ventilatory thresholds	80% of 1 repetition maximum (RM)	60% of 1RM
Time	20–60 min continuous or 30–40 min discontinuous high intensity	1–8 repetitions 2–3 sets	12–25 repetitions 2–3 sets
Type	Large muscle groups	Fast concentric for power Slow eccentric for strength	Concentric or eccentric

Specific for training in clinical practice, adapted from Ehrman et al. [12]

The second training principle we applied to our micro-plan was the principle of polarized training. According to the principle of polarized training, about 80% of the training sessions were performed below the ventilatory threshold [14]. In a practical sense, the training sessions below the ventilatory threshold were performed with a low or medium stimulation frequency (i.e., 10 Hz or 35 Hz). After these sessions, the athlete had a strong aerobic base without requiring too much time for recovery. The remaining 20% of the training sessions were performed at a high intensity to offer a strong stimulus for adaptation. We included about two to four high intensity training sessions a week, depending on the meso-plan.

### Modes of exercise

During the FES bike race, the athlete propelled himself via stimulation of the leg muscles to establish a cycling movement. Besides performing FES cycling, other exercise modalities were used during training sessions. FES cycling, arm cranking, hybrid cycling, and FES leg extensions were all included in the training programme of the athlete. All modes of exercise have their own advantages and disadvantages. By combining them, we aimed to put stress on different physiological systems of the athlete and to minimize monotony for the athlete (Table 4).

**Table 4:** Example of how the exercise modes are combined during an ‘intermediate’ week

Day	Session 1	Session 2
Monday	FES cycling 1500 m test	Electrostimulation for 20 min at low frequency
Tuesday	Arm cranking—60 min interval at 60% of maximal heart rate	Electrostimulation leg extensions for 50 min at intermediate frequency, with weights: 0.5 kg per leg
Wednesday	FES cycling 4 times 6 min, 30 W (adjust stimulation level), 10 min active rest, followed by 30 min of cycling (35 Hz)	Electrostimulation for 20 min at low frequency
Thursday	FES cycling for 90 min (35 Hz)	Electrostimulation for 20 min at low frequency
Friday	Electrostimulation leg extensions for 50 min at intermediate frequency, with weights: 0.5 kg per leg	–
Saturday	FES cycling for 90 min (35 Hz)	Electrostimulation leg extensions for 50 min at intermediate frequency, with weights: 0.5 kg per leg
Sunday	Electrostimulation leg extensions for 40 min at high frequency, with weights: 0.5 kg per leg	Hybrid or FES cycling for 120 min (35 Hz)

*FES* functional electrostimulation

FES cycling is the mode of exercise the athlete will use during the competition, which makes it a good race preparation. We stimulated the quadriceps, hamstrings, and gluteal muscles by placing a pair of electrodes on the skin above each muscle group. The specificity is a clear advantage for this mode of exercise, as it provides a specific stimulus to induce muscular adaptations. Besides the training adaptations at the level of the muscle, the cardiovascular system is challenged during FES cycling. Stimulation of the leg muscles activates the muscle pump in the leg muscles, reflected by an increase in mean arterial pressure, diastolic filling and stroke volume seen during FES cycling [15–19] and could help to improve the cardiorespiratory fitness. In addition to the favourable training effects, FES provides several health benefits for people with an SCI, like increases in muscle mass, capillary density, activity of metabolic hormones, bone mineral density and a reduction in fast fibres in the paralyzed muscles [20–22]. A last advantage is the flexibility of cycling inside or outside. The opportunity to cycle both inside on a trainer and outside on the roads is attractive for the athlete. Disadvantages of FES cycling (both for inside and outside cycling) are the preparation time needed before a training, the low power output and high fatiguability of the leg muscles. Due to the effort it takes to get prepared for an FES cycling training, the athlete preferred to do a FES cycling training for at least one and a half hours per session and about 5 sessions per week.

Electrical stimulation could also be applied separately to evoke isometric or concentric contractions in one or more muscle groups. We used this type of electrostimulation predominantly as recovery and strength training. It takes less preparation time than FES cycling and offers similar health benefits in paralyzed muscles. The athlete referred to the separate stimulation as ‘lazy training’ as he could perform a training session from his wheelchair or in bed. For the trainers, the separate stimulation was predominantly attractive due to the many possibilities for variation in the training stimulus: type of contraction (isometric or concentric), weights used as resistance, number of repetitions, duration of muscle activation, duration of rest, stimulation amplitude, stimulation frequency and pulse duration. We used the separate electrostimulation training frequently during strength blocks. Around the transition period from base training phase to the specific training phase, some weeks of intensive electrostimulation were introduced. The athlete had two to three training sessions a day during which he stimulated his quadriceps with several stimulation characteristics (10–80 Hz) to improve power output in a short time. For practical reasons, we were limited in the application of concentric contractions. Eccentric contractions are hard to control for people with paraplegia, due to the absence of muscle activity of agonist and antagonist to control and slow down the movement. The absence of general muscle tension and voluntary control increases the risk on injuries. A combination of concentric and eccentric contractions is superior to solely concentric training to increase muscle strength and power [23].

Arm cranking is another mode of exercise that was used, although seldomly, for training. Although arm cranking is the least specific exercise mode, it could assist the athlete in improving his overall fitness. The heart rates achieved during arm cranking are higher compared to FES cycling [19, 24–30] and arm cranking could function as a way to alleviate stress. Although the athlete enjoyed to push himself during arm cranking, we rarely included arm cranking sessions in our programme. The sitting position on the recumbent bike was perceived as uncomfortable to perform arm cranking exercise for long durations and switching to a hand bike was impractical in the situation of the athlete due to the time needed to change the indoor cycling set up from recumbent bike to hand bike.

Hybrid cycling is a combination of arm cranking and FES cycling. It is the most effective exercise mode to reach a high cardiac output [15–19] and oxygen consumption [17, 19, 25–30]. Besides the effectiveness to induce the cardiovascular and respiratory adaptations, muscular adaptations are triggered by the FES. Hybrid cycling is more suitable to use outside compared to FES cycling, as the arms can assist in propelling the bike and training can be continued for longer despite the fatigue in the leg muscle groups. Despite all the advantages, hybrid cycling was not perceived as comfortable by the athlete. The athlete had experiences with becoming lightheaded and having a weird feeling in his stomach during hybrid cycling. Therefore, hybrid cycling was avoided in the programme.

### Performance assessment

Training is all about giving the minimal stimulus while accomplishing maximal performance. A successful training programme will be reflected by performance improvements. A method to monitor the performance of the athlete is via objective measures. The athlete performed a 1500 m test on the smart trainer to monitor his performance in a controlled situation. We chose a 1500 m instead of a 1200 m, as differences in programme setting might lead to slightly higher resistances during the competition. When the weather allowed us to go outside, the athlete performed a trial outside to monitor his performance in a real-life situation.

**Fig. 1:**
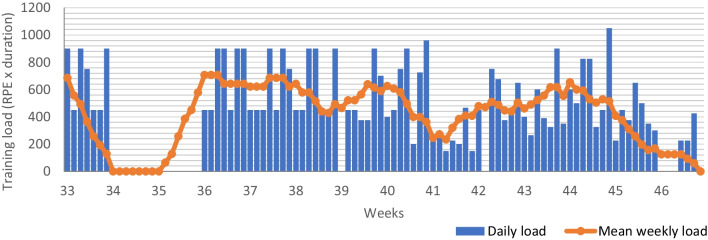
Daily training load and mean weekly training load for week 33–46 (base training)

The objective measures can be combined with subjective measures to monitor whether the prescribed stimulus evoked the desired response. The athlete performed around 12 training sessions a week, so we were wary of overtraining. By using six subjective measures to monitor the athlete, we aimed to detect early signs of overtraining before performance declined. First, we monitored the weekly training load using the surrogate TRaining IMPulse (TRIMP) score. The surrogate TRIMP score combines the external and internal load [31], which makes it suitable to check whether high intensity weeks were perceived as intended. Rate of Perceived Exertion (RPE) (from 0 = no effort to 10 = maximal effort) was scored by the athlete for each training session and combined with the duration of the training session to quantify training load. An example of the visual representation of the training load can be found in Fig. 1. Second, the surrogate TRIMP score was used to calculate training variability (monotony = daily training load/standard deviation) and strain (stress score = weekly training load*monotony). A low training variability at a high load might contribute to the development of overtraining syndrome (inaccurate recovery from repetitive intense training [32]) and a high training strain is found to correlate with a high prevalence of banal infections [33].

## Results

Regarding performance assessment using objective measures, we were able to collect data from the 1500 m tests inside, rides outside and the three Cybathlon Global Edition 2020 races. Although the athlete consistently reduced his finish time of the 1500 m test on the indoor trainer during first months, we also detected some sudden changes in performance (Table 5). The cycling tests outside were performed less often, due to the dependency on weather conditions and the time needed to prepare the bike for outside riding. After hearing about the change in the racing format in spring 2020, from oval track to indoor trainer, the tests on the indoor trainer were even more prioritized. Large variability is present between trials (Table 6).

**Table 5:** Times of a 1500 m inside as a preparation for the Cybathlon Global Edition 2020

Date	Trial 1				Trial 2			
	Time	Mean power (W)	Max power (W)	Power just before finish (W)	Time	Mean power (W)	Max power (W)	Power just before finish (W)
28-11-2019	0:04:47	34	62	25	0:04:52	31	50	23
21-12-2019	0:04:46	34	66	24	0:04:59	29	53	20
22-12-2019	0:04:40	35	51	27	0:04:55	30	44	24
23-12-2019	0:04:33	39	80	27	0:04:48	33	52	24
28-12-2019	0:04:46	33	76	23	0:05:07	26	41	18
6-1-2020	0:04:35	39	90	26	0:04:46	33	65	22
9-1-2020	0:04:34	38	82	27	0:04:48	33	63	23
21-1-2020	0:04:46	33	82	20	0:05:01	28	54	15
14-2-2020^a^	0:04:20	35	74	24				
28-2-2020	0:04:20	34	65	21	0:04:38	28	54	20
29-2-2020	0:04:09	40	86	26	0:04:18	35	67	25
1-3-2020	0:04:13	38	91	28	0:04:38	33	50	18
3-3-2020	0:04:23	33	77	19				
12-4-2020					0:04:13	34	62	22
29-4-2020	0:04:25	33	73	21	0:04:37	28	64	18
1-5-2020	0:04:25	33	51	27	0:04:32	31	42	23
3-5-2020	0:04:17	37	55	28	0:04:29	32	48	24
4-5-2020	0:04:49	25	46	16				
15-5-2020	0:04:14	38	62	29	0:04:40	28	38	20
16-5-2020	0:04:22	34	55	23	0:04:25	33	54	24
25-5-2020	0:04:24	34	55	27	0:04:37	29	43	22
11-6-2020	0:04:47	34	46	28				
15-6-2020^a^	0:04:42	35	63	25	0:04:50	32	52	22
16-6-2020^a^	0:04:34	39	61	31	0:04:21	35	49	27

Mean power, maximal power and power just before finishing were collected during two trials. Missing values were the result of technical problems or uncomfortable feelings during the trial

^a^Represents a change in the bike set up, stimulation pattern or settings in the trainer

**Table 6:** Times of a cycling test outside as a preparation for the Cybathlon Global Edition 2020

Date^a^	Trial 1			Trial 2			General				
	Time	Distance (m)	Average speed (km/h)	Time	Distance (m)	Average speed (km/h)	Location	Wind speed (km/h)	Wind direction	Bike	Comments
7-10-2019	00:03:15	490	6.4	00:02:45	450	4.5	Lyon	17	N	Pulse	Straight course, slightly upwards, soft tires
20-10-2019	00:09:13	1890	12.3	00:08:05	1430	10.6	Hoogkarspel	14	NO	Sander	1st trial headwind, 2nd trial tail wind, straight course
30-11-2019	00:09:16	1960	12.7	00:08:55	1420	9.6	Hoogkarspel	11	W	Sander	1st trial headwind, 2nd trial tail wind, straight course
3-1-2020	00:01:42	290	10.1	00:04:19	780	10.9	Amsterdam Bosbaan	30	W	Pulse	1st trial headwind, 2nd trial tail wind, straight course
4-3-2020	00:07:00	690	5.9	12:00:00	440	8.5	Amsterdam athletics	28	WSW	Pulse	400 m athletics track, synthetic rubber
12-3-2020	00:02:08	260	7.3				Amsterdam only friends	46	WSW	Pulse	400 m circle, soft underground, troubles with stimulation
27-5-2020	00:10:35	1910	10.8	00:12:45	1900	9	Hoogkarspel	25	NNW	Sander	Gear too low, straight course
3-6-2020	00:10:16	1920	11.2	00:04:11	740	10.6	Hoogkarspel	25	N	Sander	Straight course

Time, distance and average speed were collected during two trials. Information about location, wind speed, wind direction, type of bike, and other comments, is relevant for interpretation of time and distance between dates. Missing values were the result of technical problems or uncomfortable feelings during the trial.

^a^Represents a change in the bike set up, stimulation pattern or settings in the trainer

The penultimate race, the Cybathlon Global Edition 2020, took place on the 13th of November. The last 1500 m indoor cycling trial was the fastest (Table 7). Race times were 2 min and 53 s during the first, 2 min and 41 s during the second and 2 min and 40 s during the third trial. Overall, this resulted in the first place during the FES cycling race during the Cybathlon Global Edition.

**Table 7:** Finish time, power output and split times over the three Cybathlon Global Edition 2020 races

	Finish time (s)	Split time (s) 200 m	Split time (s) 400 m	Split time (s) 600 m	Split time (s) 800 m	Split time (s) 1000 m
Race 1	173	30	56	84	113	144
Race 2	161	32	58	82	108	134
Race 3	160	34	61	85	110	135

**Fig. 2:**
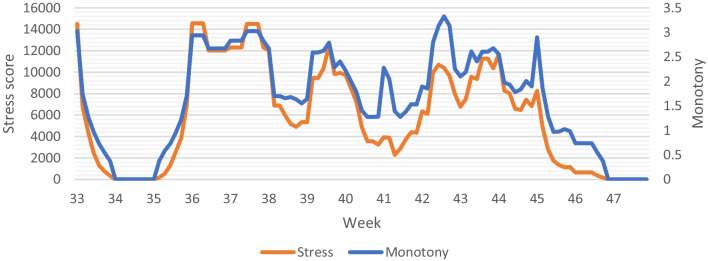
Visual representation of the monotony and stress score for week 33–46 (base training)

The subjective measures are visualised in Figs. 1 and 2. The sudden drops in training load in week 34 and 35 are clearly recognizable in the figures. Total training load did not increase as originally proposed in the training programme. Variability, represented by monotony, and strain, represented by the stress score, fluctuate over the 15 weeks.

## Discussion

Although the plan was formulated at the start of the year, it was continuously subject to changes. During the first months of the preparation period, the health status of the athlete did not allow him to train as much as he wanted. The skin of the athlete’s upper legs, where the electrodes are placed, was irritated and the skin condition became worse after applying stimulation. In consultation with a health specialist, the athlete stopped stimulating his leg muscles. After a few weeks without stimulation his skin condition improved, but never fully recovered. From that moment, we were continuously searching for the right balance between sufficient FES application to evoke muscle adaptations while minimizing skin irritation. Besides the skin problems, the athlete has ongoing bladder problem. A common problem in those with SCI with over 65% of individuals suffering at least once from urinary tract infections after discharge from initial hospitalisation [34]. Bladder infections were already a problem for the athletes before he started training with FES and we have no evidence that training was increasing their prevalence or intensity. Many of the bladder infections were light and had minor effects on the training sessions of the athlete. Unfortunately, one infection caused fever and forced the athlete to quit training for a few weeks. After this period, we included some weeks of slowly building up training load before continuing with the original training programme.

The second change in the plan was caused by the race date being rescheduled. The Cybathlon Global Edition 2020 was postponed by at least four months, so we decided to go back to our base training phase. The health problems of the athlete had caused some delays in our previous base training phase, so we could use the extra time to further improve the endurance capacities of the athlete. Besides adjusting the training programme, it is important to give the athlete some mental support to cope with the situation. The athlete had strong intrinsic motivation, so it was mainly our task to prevent him from overtraining. Involving the athlete while making a new plan and setting new goals gave him understanding behind the decisions made.

Finally, we could start testing the smart trainer on which we would perform the FES bike race a few weeks before the racing day. Riding on the smart trainer was slightly different from riding the bike outside, so we needed to make some adjustments to our racing set up and strategy. Training sessions during the taper period were therefore filled with testing and adjusting the racing set up, practising with allowed starting speed of maximum 10 km/h, finding the right cadence, fine-tuning the level of stimulation during the race and choosing the right gearings for optimal performance. The continuous adjustments made to keep the athlete on track of the original training program emphasize the importance to keep in close contact with the athlete.

### Objective measurements

Performance was monitored using a 1500 m test inside and a cycling test outdoors. The performance measures are ideal to check the current status of the preparations, but they are also important to keep the athlete and team motivated. Outside, the low power output and high fatiguability in combination with obstacles on the road made it difficult to do FES cycling for longer than 2000 m outside. We did not stick to a certain distance outside, as the conditions in each trial differed tremendously. Covering as much distance as possible was a good way to motivate the athlete, but the resulting measurements contain too much confounding variables to evaluate performance. Training inside on a smart trainer ensured a controlled setting for the 1500 m test and to perform interval and endurance training sessions. The slope could be adjusted during a training session to reduce resistance and allow for cycling for longer durations (one hour or longer) despite the low power output. Unfortunately, the performance of the athlete varied quite considerably (Table 5). Thus, to be able to detect changes in performance over time in spite of this random variation, we advise the 1500 m test be performed on the smart trainer on a weekly or biweekly basis. Moreover, we suggest to combine the objective measures with other performance related observations, for example leg circumference and skinfold thickness as a surrogate for muscle mass [35], slope and power output during an endurance training session, decrease in power output during an interval training, weights used during a leg extension (strength) and the stimulation needed to evoke contractions (reactivity of muscle). Additionally, daily evaluation of the resting heart rate would provide an objective measure of overtraining [36]. We aimed to implement this in the programme, but we did not collect sufficient data.

### Subjective measurements

The TRIMP score, variability and strain were monitored to quantify how the athlete reacted on the programme. The mean weekly training load decreased during the base training period, which is not what we intended. However, the above-mentioned problems we encountered forced us to change the programme.

For a complete picture of the athlete’s reaction to the training programme, we included daily questions about readiness to train (from 0 = not ready to 10 = completely ready) and perceived general fitness (from 0 = not fit at all to 10 = very fit) and questions related to SCI (perceived spasticity and nausea feelings in a scale from 0 = not present to 10 = strongly present). The combination of readiness to train and general fatigue has been found to be a sensitive indicator to detect functionally overreached athletes [37]. Additionally, by monitoring spasticity and nausea on a regular basis, it might be possible to detect their relationship with training load or stimulation intensity. Unfortunately, the athlete had problems filing in these questions which limited our data acquisition.

The rationale behind all the subjective measures is evident, however, in practice, the athlete had difficulties with the daily scoring report. For example, rating his RPE following a training session could not be scored, as the athlete couldn’t feel whether his legs were tired or not. Moreover, the density of information the athlete could share is restricted to the questions asked. In our opinion, the best option to monitor the athlete is to join training sessions regularly, while observing and listening to the athlete. Rating of subjective measures should be seen as a helpful addition to the direct contact with the athlete, as they might provide data that is suitable for making visual representations.

### Athlete perspective

My name is Sander Koomen, and in 1994, at the age of 21, I had an accident in a bicycle race and broke my spinal cord. From that moment I don’t have any control over my body under the level of T6, the lowest I can feel is the caudal side of my chest bone.

Somewhen in 2010, I was asked for the first time to be a part of a research in electrostimulation and the third one I participated in was a study with a BerkelBike (BerkelBike Connect, BerkelBike, Sint-Michelsgestel). In that study, half of the participants would train on a normal hand bike and half on a BerkelBike with FES stimulation. I was the lucky one to be drawn for the BerkelBike. That research made me have a lot of interest and fun while my legs being stimulated, so I applied for a BerkelBike myself (with the help of healthcare). I tried to train once every week on an indoor trainer for 90 min. Not very structural, but just for fun to see my legs work again.

Somewhen in 2017, I was again contacted for research, this time it was the PULSE Racing team and the goal was the 2020 Cybathlon Global Edition 2020. From that moment it all became much more serious. The first months were mainly focused on overall physical health. Then slowly the focus shifted towards training the leg muscles. I am lucky to have a BerkelBike at home and having a Compex trainer (Compex Sport, Compex, Guildford) as well. A great advantage of the Compex trainer is that I can train my muscles while sitting in my lazy chair and I don’t need much time to prepare for the training.

Over time the training programme became more and more fine-tuned, and the intensity further increased. In 2019, I started to see the muscles grow, which was a huge mental boost for me. As we were training for the Cybathlon, we sometimes did outside runs too. We saw a steady increase in the distance I could travel. In the end of 2019, I was able to make two runs of 2 km, which would be enough to make the full distance at the Cybathlon Global Edition 2020, one of the main goals. For me, at this point, the time and effort invested started to accumulate, resulting in several health issues. The training level was adjusted to my problems to prevent losing training time again. Sadly enough, at that point the Cybathlon Global Edition 2020 was already postponed. I was looking forward to some rest when the race would be over. Again, I felt lucky to have all training possibilities at home, so the COVID-19 didn’t have much influence on the training schedule, I was only missing the social part of the research. In October 2020, the Cybathlon staff found a way to let the event happen. None of us expected we could win; we all knew a podium was possible but winning was not expected at all.

Thanks to all members of PULSE racing for the fantastic time you made me have and thanks for the Cybathlon for finding a way to make the event happen despite all the problems.

## Conclusion

In conclusion, physical training was the primary focus of PULSE Racing during the last preparation phase for the Cybathlon Global Edition 2020. To optimize the physical state of the athlete, training objectives were defined, and training sessions were planned at macro, meso and micro level. The main challenge was to keep adjusting the plan to the current state and needs of the athlete. Monitoring of the training adaptations helped in personalizing the plan. However, the athlete was the expert on his own needs throughout the duration of the training. It is the athlete in the end who won the gold medal. To continue making progress, we advise to keep improving the standardization and frequency of collecting both objective and subjective data, which will allow for continuous adaptation of the training plan to meet the needs of the athlete and reach the pre-set goals.

## Data Availability

The datasets used and/or analysed during the current study are available from the corresponding author on reasonable request.

## Abbreviations

AIS: ASIA impairment scale
COVID-19: COronaVIrus Disease 2019
FES: Functional Electrical Stimulation
FITT: Frequency, Intensity, Time, Type
SCI: Spinal Cord Injury
SMART: Specific, Measurable, Achievable, Relevant, Time-bound
TRIMP: TRaining IMPulse
